# Teleophthalmology for First Nations Clients at Risk of Diabetic Retinopathy: A Mixed Methods Evaluation

**DOI:** 10.2196/medinform.3872

**Published:** 2015-02-23

**Authors:** Julie Kim, D Dean Driver

**Affiliations:** ^1^University of VictoriaVictoria, BCCanada; ^2^Inter Tribal Health AuthorityNanaimo, BCCanada

**Keywords:** Ophthalmology, Diabetic Retinopathy, Telemedicine, Mobile Health, underserved populations

## Abstract

**Background:**

Access to health services is a particular challenge for First Nations (aboriginal Canadians) communities living in remote or underserviced areas. Teleophthalmology can provide them with the same level of retinal screening services provided to those in urban centers. This screening can lead to the identification of high-risk individuals who can then be monitored and receive treatment related to their diabetes or other health issues.

**Objective:**

The intent was to develop, implement, and evaluate a service delivery model for teleophthalmology screening and follow-up for at-risk and diabetic First Nations clients on Vancouver Island, British Columbia, Canada.

**Methods:**

A highly consultative, culturally appropriate, and collaborative approach was used to develop and deploy a teleophthalmology service delivery model to First Nations communities. This project was evaluated with regard to utilization and operational costs. Also, clinicians and team members involved in the teleophthalmology project provided assessments of the teleopthalmology quality, productivity, and access. Health providers in First Nations communities provided their perceptions of areas of improvement for the remote retinal screening services, areas where expansion of services could be offered, and opportunities to increase client education and health promotion.

**Results:**

All 51 First Nations communities on Vancouver Island expressed interest in receiving teleopthalmology services. During the 1-year project, teleopthalmology clinics were held in 43 of 51 communities on Vancouver Island. During these clinics, 524 clients were screened and 140 of those clients were referred to a general ophthalmologist, family doctor, retinal specialist, optometrist, or other provider. Ratings of teleopthalmology system quality, information quality, service quality, and system usage were positive. Satisfaction with the teleopthalmology project was high among clinicians involved with the project. Satisfaction was also high among health providers in First Nations communities, with clinic scheduling identified as a potential area of improvement moving forward. The average cost savings per client, taking project costs into consideration, was calculated to be CAN $28.16, which was largely due to the elimination of client travel costs.

**Conclusions:**

Teleophthalmology was a welcome addition to health services by the First Nations communities on Vancouver Island, as evidenced by the 100% rate of interest from those communities. There was no evidence of dissatisfaction by clinicians involved in the teleopthalmology project or by First Nations community health providers. The now-operational teleopthalmology program is a testament to the early success of the project.

## Introduction

### Overview

Aboriginal peoples living in Canada are among the highest risk populations for diabetes and related complications [1].

For First Nations communities, which are most often remote and geographically isolated, availability of primary health care is often reduced, and access to secondary and tertiary care can be challenging or non-existent. This includes access to eye-related services such as ophthalmology and optometry, which are important for at-risk and diabetic individuals.

Teleophthalmology enables remote populations to access retinal screening services provided more readily in urban centers. This screening can lead to the identification of high-risk individuals who can then be monitored and receive treatment related to their diabetes or other health issues.

Mobile screening initiatives for First Nations and aboriginal populations have previously been conducted and evaluated in Canada. A retinopathy remote screening pilot project among Quebec First Nations populations found positive outcomes with respect to satisfaction among community members and users, and financial and societal costs [2]. In Alberta, a mobile screening program for diabetes complications in aboriginal communities was found to be effective with respect to clinical outcome measures [3]. Mobile diabetic retinopathy screening among isolated First Nations people in remote Ontario has been shown to be cost-effective [4]. Teleophthalmology screening for diabetic retinopathy through mobile imaging units in five Canadian provinces was shown to efficiently lower barriers to screening and create new screening opportunities for a large number of known diabetic individuals who would not have been screened in the traditional health system [5].

### This Initiative

The teleopthalmology project began in November 2008 and was officially launched on May 14, 2010. The project was led by the Inter Tribal Health Authority (ITHA) in British Columbia (BC), in collaboration with the Vancouver Island Health Authority (VIHA). The project concluded in July 2011, at which time it became and still remains an operational program.

### Objectives

The objectives of the teleophthalmology project were to develop and implement an effective teleophthalmology service model, which included an accessible and integrated mobile retinal screening and tracking service. This teleophthalmology service model would be implemented for high-risk populations (specifically, First Nations peoples and diabetics) in rural and remote populations at risk of retinopathies. A target of 600 people at risk of retinopathy was set for screening. It was anticipated that the service model developed and used for the teleophthalmology project would provide a basis for a broader provincial program. 

The project focused on retinal health and eye disease, and was not intended to replace routine visits to optometrists. In fact, referrals resulting from teleophthalmology visits would include referrals to optometrists.

## Methods

### Framework

The Canada Health Infoway Benefits Evaluation Framework [6] provides a high-level evidence-based model to guide evaluations of health information systems. It includes six main dimensions of system quality, information quality, service quality, use, user satisfaction, and net benefits. The Benefits Evaluation Framework was referred to as a guide to determine the evaluation design and to identify measures.

### Recruitment, Communication, and Community Engagement

Matters of communication and community engagement were particularly critical to ensure awareness of vision-related disease among diabetics in First Nations communities and acceptance of the teleophthalmology project. Adherence to, and respect of, First Nations’ cultural values were considered to be the most important aspects and success factors of the project.

The first 5 months of the project were dedicated to implementing a community engagement strategy. The teleopthalmology project team sent letters to all 51 First Nations communities on Vancouver Island to determine the communities’ interest in a short presentation about the project.

The communities were invited to attend project launch meetings. Representatives from all communities attended these meetings, which served as informational sessions that invited participation in the teleopthalmology project. Attendees included chiefs, band members, community nurses, and representatives from both VIHA and ITHA. These meetings adhered to cultural protocols, and were widely publicized by the media, sponsors, and ITHA. Key messages pertained to awareness about the project, the importance of retinal screening, and implications for those suffering from diabetes.

After the meetings, representatives from First Nations communities that were interested in participating in the project were welcome to contact the teleophthalmology project team. Clinic dates and times for communities were determined based on the communities’ availability and preferences.

As well, a letter of intent was also sent to the British Columbia Association of Optometrists to its members of the teleophthalmology project. This letter provided details about the project, and also explained that the project was not intended to replace optometrist visits or discourage clients from making routine visits with optometrists.

### Project Resources and Governance

#### Overview

The Inter Tribal Health Authority was responsible for overall management and direction of the project. Various ITHA team members developed materials, put together the community engagement strategy, developed and delivered communications with stakeholders, and addressed logistical and technical matters. A Telehealth Project Office was instituted within ITHA, and the provincial Telehealth officer oversaw the project.

A medical advisor and clinical advisory group were put in place for this project. They provided medical expertise, community engagement advisement, and acted as a sounding board to ensure that the project operated in a culturally appropriate manner with the First Nations communities. The clinical advisory group consisted of administrators, ophthalmologists, First Nations leaders, various health care providers, ITHA leaders, and teleophthalmology project team members. One key representative from each of the three language groups across Vancouver Island was invited to join the clinical advisory group to guide and protect their interests and convey information to and for their communities.

#### Identification of Target Clients

Potential clients were initially identified through a combination of activities and information sources, and in cooperation with the existing chronic disease management (CDM) program and health services providers. The participating First Nations communities provided ITHA with lists of individuals in their communities who were diabetic, individuals previously diagnosed and treated for type 1 or type 2 diabetes, individuals at risk of diabetes, or individuals with a family history of diabetes.

These individuals received phone calls from the ITHA team to schedule individual screening appointments during clinics to be held in their communities. Other individuals also learned about upcoming clinics in their communities through local health care providers and by word of mouth. The clinical advisory group recommended that an open door policy exist for the screening clinics, and individuals who self-referred to the clinics also received services.

### Development of Teleophthalmology Service Delivery Model

#### Overview

The teleophthalmology project involved the implementation of a service delivery model, including the technology to deliver services. The service delivery model arose from a detailed workflow analysis directed by the clinical advisory group and the medical advisor. A comprehensive clinical procedure and protocol manual was developed by ITHA to define and standardize how the mobile teleophthalmology clinics would be conducted. It identified all processes, materials and equipment, and roles involved. The manual included the teleophthalmology service delivery model.

#### Description

##### Overview

This teleopthalmology service delivery model provided early retinopathy detection services to residents of rural and remote communities, and utilized a proven pathway for effective treatment of retinopathy that can reduce incidence, as well as social and economic impacts, of blindness and vision impairment [7].

The service delivery model provided the following set of services: retinal screening, triage, treatment, and tracking. A description of the activities and workflow are discussed below; following that is a description of the technology and equipment.

A team of two First Nations technicians and a trained teleopthalmology eye care nurse travelled to the remote First Nations communities within the geographic region of the VIHA. They travelled in a truck that was dedicated to the teleopthalmology project; the truck was fully outfitted with the equipment required to hold the clinics.

Wherever possible, clinics were held within existing health centers in the communities. In some cases, they were held in community centers or gymnasiums, with provisions made for client privacy during appointments. Clinics typically operated from 8am until 4pm, varying based on travel schedule and client volume.

##### Client Appointments

The nurse registered individuals upon their arrival to the clinics. In the waiting area, clients were given pamphlets that explained the importance of the teleopthalmology project. They were also given small MP3 players with which to listen to diabetes education content while awaiting their appointments.

**Figure 1:**
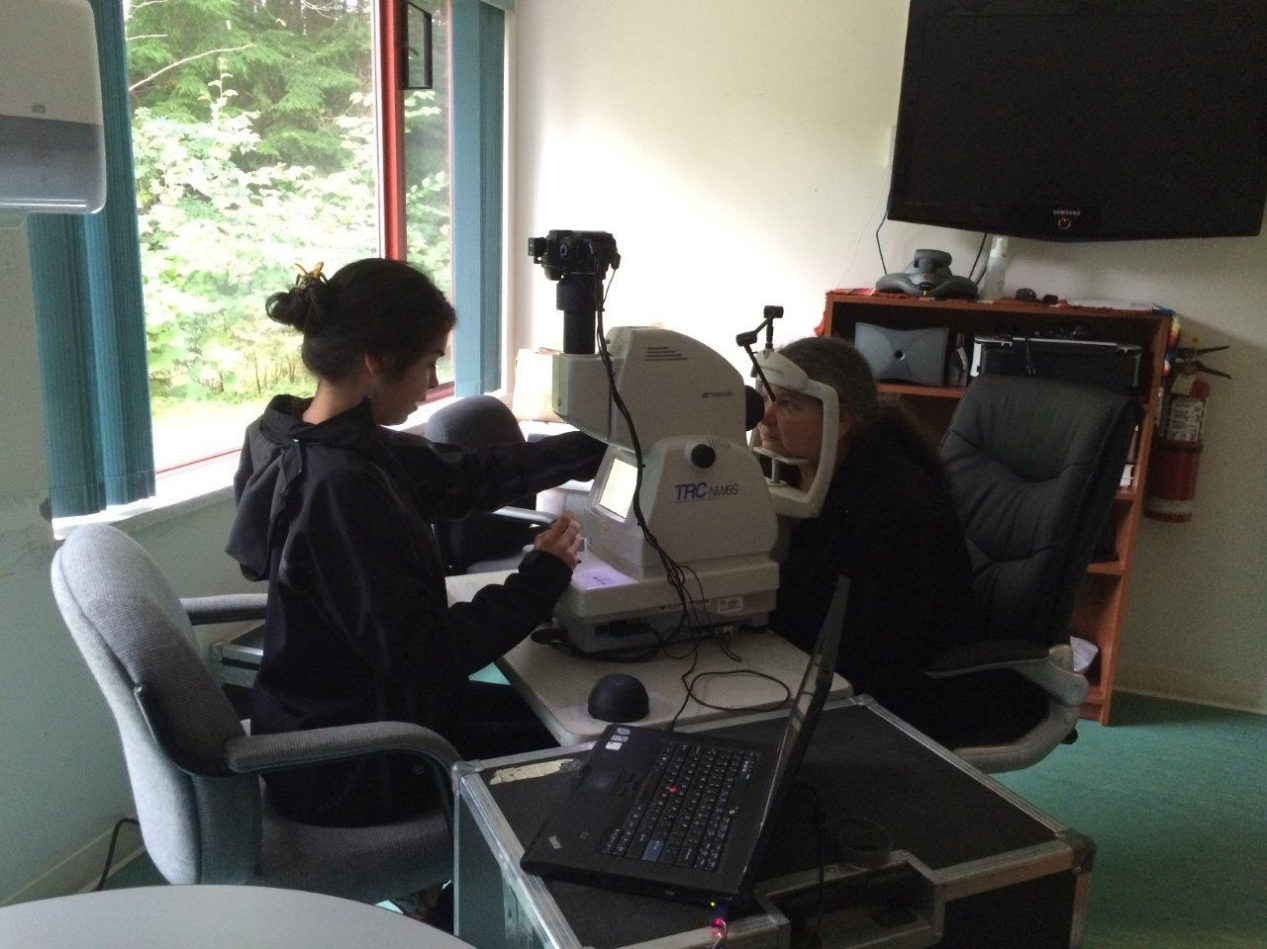
Teleophthalmology screening appointment.

The clients were then assessed for details of visual function, intraocular pressure, and three-dimensional digital photographs of the anterior and posterior segments of the eye. Alcaine drops were used for freezing prior to intraocular pressure testing and Tropicamide drops were used for dilation. Pilocarpine drops were kept in case of a close angle glaucoma reaction. Drops were administered by the nurse. Figure 1 shows a teleopthalmology screening appointment.

Client data collected for registration and screening included: demographics, an abbreviated medical history, tonometry, and visual acuity results.

##### Medical Assessments

**Figure 2:**
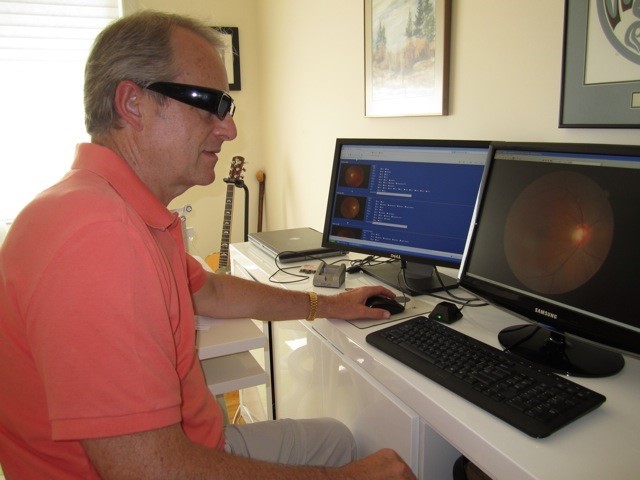
Grading physician review of results.

The technicians securely uploaded images to the ITHA Data Centre for subsequent assessment by either an ophthalmologist in Comox, BC or a retinal specialist in Victoria, BC. Their documentation and reports included recommendations for follow-up examinations or specific additional tertiary examination and treatment. Figure 2 shows how grading physicians viewed results.

##### Follow-Up

Follow-up communications and appointments proceeded based on the grading assessments and results. The teleopthalmology project at ITHA effectively operated as a satellite office for the ophthalmologist and retinal specialist by scheduling follow-up appointments with other eye specialists on Vancouver Island based on the type of referrals made during the grading of the retinal images.

All clients were contacted after their appointments regardless of the outcomes of their assessments. They were mailed a copy of the teleophthalmology report unless mailing addresses were unknown. In those cases, reports were sent via fax to the clients’ local community health units. The clients’ physicians also received a copy via mail.

ITHA produced a weekly summary for the clinical advisory group. It combined the standard reports used by the teleopthalmology project team (ie, Client Summary Report, the Follow-Up Report, the Diabetic Retinopathy Report, and the Referral Report).

##### Technology and Equipment

Mobile units contained program equipment and disposable clinic supplies. These included a retinal camera, peripheral devices, laptop, image capture software, and travel containers.

The camera was a Topcon NW6-S Retinal Camera, which weighed 89 lb including the case. A truck lift was required for the vehicle, and a two-person lift was required where planes, barges, and boats were involved. Setup time was 30 minutes for the camera, table, laptop, and chair.

**Figure 3:**
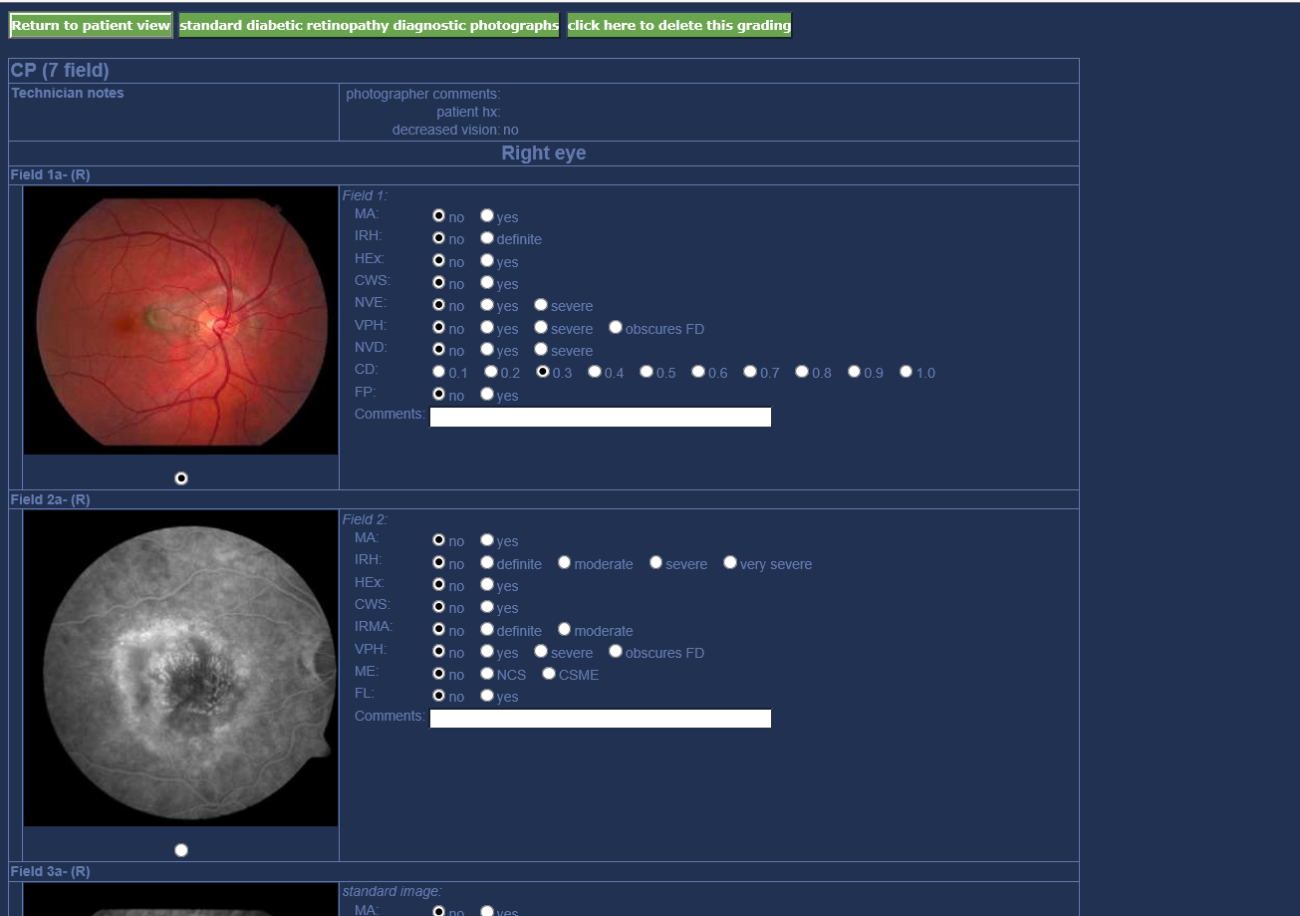
Screenshot of grading system.

The software used by mobile screening clinics was a combination of IMAGENet [8] and the Secure Diagnostic Imaging (SDI) application [9]. IMAGENet enabled the capture of images and the SDI application provided a standardized format for the grading physicians. Seven-field stereoscopic imagery was used as the standard to build complete image sets of each eye. This provided the grading physicians with a three-dimensional high-resolution perspective of the eye similar to a live exam. Figure 3 shows the user interface. The SDI application also produced a final report that was used as the basis for any subsequent referrals. Client data was then synchronized with the Intrahealth Profile Electronic Medical Records System for more detailed administrative and clinical management [10]. Intrahealth Profile was also used to capture client demographics upon registration, record measurements, document screening appointments, create client letters, and manage follow-up and referrals.

The retinal images that were captured during the clinics were securely uploaded, stored, and backed up on a daily basis. This was an important consideration for First Nations in BC, as autonomy and ownership of First Nations data is foremost in the adoption of new health information systems. The First Nations Principles of Ownership, Control, Access and Possession (OCAP) apply the concepts of self-determination and self-governance to research, statistics, and information involving First Nations communities. The Inter Tribal Health Authority ensured that the project strictly adhered to OCAP principles.

All data was stored at the ITHA data center, a First Nations operated data storage facility located in BC. The Inter Tribal Health Authority is a First Nations organization; it controls, stores, and owns the data collected in the project. It is on Snuneymuxw territory enabling the physical possession of the data on First Nations land consistent with the OCAP protocol.

### Implementing the Service Delivery Model

Training and capacity building for the teleopthalmology project include three unique aspects: specialized one-to-one training, job shadowing, and capacity building among First Nations youth. Detailed logs by clinic members were kept and reviewed to continually improve project operations.

Specialized one-on-one training for team members with an SDI and teleophthalmology expert addressed clinical processes and client care, retinal image photography, equipment maintenance, secure image transmission, mock clinics, and cultural protocols.

Job shadowing took place in the offices of the grading physicians—the ophthalmologist in Comox, BC and the retinal specialist in Victoria, BC. During these work experiences, the nurses and technicians were able to see how the clinics operate, how retinal scans were taken, and how clients were treated in busy urban practices.

One of the known deficiencies in the aboriginal health field is the markedly low level of First Nations people trained in health care professions. The teleopthalmology project provided a capacity building opportunity to three First Nations youth and supported their continued education and work experiences.

Each member of the teleopthalmology team maintained detailed logs of each clinic that was delivered. These logs were free-form, hand-written notes about team members’ experiences and lessons learned from each clinic. They pertained to logistics of booking the clinics, equipment, perceived assessments of the clinics, and observations about cultural protocol. The hand-written notes were converted to electronic format by the technicians upon returning to ITHA offices. They were all incorporated into a Microsoft Word document that continued to be updated with information to assist in the continual improved provision of the clinics.

### Evaluation Approach

#### Overview

The evaluation for the teleopthalmology project involved multiple data collection sources and activities. These were: utilization measurement, system and use measurement, community health provider feedback, and operational cost measurement.

The ITHA teleopthalmology team conducted analysis for the various data collection activities. The primary author of this paper worked with the second author (the ITHA teleopthalmology team leader) to synthesize this analysis, conduct additional analysis, and develop the paper.

#### Utilization Measurement

Individual client records from the SDI database were summarized and compiled for analysis. Reports from the SDI program were used to produce summaries from the retinal screening clinics. These included the number of clients screened, the number of clients referred, and the results of diagnoses by the grading physicians.

#### System and Use Measurement

The System and Use Survey (S&U Survey) was developed by Canada Health Infoway as a tool to assess quality and use of health information technology systems and also to flag obstacles to the adoption of systems and the realization of net benefits from them. The S&U Survey was customized to consist of 25 questions and was designed to determine initial reaction to the screening solution in relation to quality, productivity, and access. Multimedia Appendix 1 provides the S&U Survey used for the teleopthalmology project.

The S&U Survey was developed in a web-based format using SurveyMonkey and sent via email to clinicians involved in the teleophthalmology project. Clinicians were also given the option to request and submit a paper version of the survey.

Survey results were extracted and interpreted by the ITHA project team using simple univariate analyses.

#### Community Health Provider Feedback

The Community Health Provider Feedback Survey was developed by ITHA for this project. The survey, which was administered using SurveyMonkey, consisted of 10 questions. It was designed to solicit feedback on the retinal screening clinics from those who deliver health care in each of the communities. It asked respondents to identify areas where the retinal screening service could improve, to identify areas where expansion of services could be offered, and to identify opportunities to increase client education and health promotion. Multimedia Appendix 2 provides the Community Health Provider Feedback Survey.

The teleopthalmology project team sent a Web-based survey via email to 22 community health care providers from communities that had received retinal screening clinics during the past 11 months.

As with the S&U Survey, results from the Community Health Provider Feedback Survey were extracted and interpreted by the ITHA project team using simple univariate analyses.

#### Operational Cost Measurement

Operational costs to support the delivery of the retinal screening clinics were examined and compared to what could be identified as pre-teleopthalmology costs for retinal screening.

Travel costs for each of the First Nations communities were tabulated based on then-current Non-Insured Health Benefits (NIHB) Health Canada reimbursement schedules. Additionally, Medical Services Plan (MSP) base costs for Optical Coherence Tomography (OCT) scans, general practitioners, and specialist referrals were applied to the relevant points in the service delivery model.

A value for average client travel cost was provided by the NIHB program. Travel costs for each First Nation were based on the distance to the nearest ophthalmologist, calculating the cost of a return trip to and from that ophthalmologist for each client. An additional 15% of travel costs were added for those cases where an escort for the client was required. This 15% value was recommended by the NIHB team, based on historical travel records.

## Results

### Screening Communities and Participants

Teleophthalmology screening was conducted in 43 of the 51 First Nations communities on Vancouver Island in the first year of operation (the evaluation period). These First Nations communities are listed in Multimedia Appendix 3, including volumes for screening and referrals by community. Although all 51 First Nations communities expressed interest in the teleopthalmology project, resource and time constraints allowed for the deployment of clinics in 43 of these communities during the first year.

In these 43 communities, 524 diabetic and at-risk individuals were screened. Table 1 shows the breakdown of clients screened by age and gender. Of the 524 at-risk individuals who were screened in the first year, 140 were referred for additional treatment. This represents 26.7% of all clients screened; these screened clients were from 30 of the 42 First Nations where screening clinics were held. In communities where there were referrals, between 12% and 100% of clients who were screened were referred.

Table 2 shows the overall breakdown of the type of referrals that were made following the delivery of the retinal screening clinics. It shows that the majority of referrals were for general ophthalmologist services.

The retinal screening revealed a variety of eye disease and health problems apart from diabetic retinopathies. Other conditions that were diagnosed included cataracts, glaucoma, hypertension, macular degeneration, and other vascular diseases.

**Table 1:** Breakdown of teleopthalmology clients screened (n=524).

Teleophthalmology clients		n (%), mean or total years
Total female clients, n (%)		296 (56.5%)
Total male clients, n (%)		228 (43.5%)
**Average age, years**		55
	Female	54
	Male	57
Youngest client, years		9
Oldest client, years		93

**Table 2:** Number of teleopthalmology clients per referral type (n=140).

Type of referral	n (%)
General ophthalmologist	102 (72.9%)
Family doctor	14 (10.0%)
Retinal specialist	12 (8.6%)
Optometrist	10 (7.1%)
Other	2 (1.4%)

### System and Use Measurement

#### Overview

Of the eight participants invited to take the S&U Survey, seven individuals responded. All seven used the electronic format of the survey. These participants were teleopthalmology team members, including the system administrator.

#### Overall User Satisfaction

Five of the seven respondents were highly satisfied with the system in terms of ease, functionality, quality of information given, and quality of services provided for the system. Two respondents were moderately satisfied.

Six out of seven respondents strongly or moderately agreed with the following statements: “the system improves my productivity”, “the system improves the quality of care I can provide”, “the system makes my job easier”, “the system enhances our ability to coordinate the continuity of care”, and “the system enhances the efficiency of ordering retinal screening referring clients for onward treatment client follow-up”. For all of these, one respondent (the system administrator) indicated that these were “not applicable”.

For the following two statements, five of the seven respondents indicated that they strongly or moderately agreed and one indicated that she/he moderately disagreed: “the system improves our sharing of client information amongst providers”, and “the alerts, reminders and order set features (ie, support tools, reporting) improve the quality of my decision-making”.

Three of the seven respondents answered an open-ended question about aspects of the system that they would change, and if so, what they would be. These included better integration of the SDI tool with other health systems, more flexible reporting, the addition of mobile units to increase speed and efficiency of screening, and additional information provided in reports.

Two of the seven respondents answered an open-ended question about describing experiences with the system where it had supported the provision of care. One of the respondents, the system administrator, described witnessing the expedited manner in which providers conveniently worked together to identify conditions requiring immediate attention and avoiding dire clinical consequences. The other respondent attested to the timely and effective manner in which specialist care was provided to clients.

#### System Quality

Of the seven respondents, five rated the system as being highly acceptable and two indicated that it was moderately acceptable.

All seven respondents strongly agreed with the statement: “the system is reliable in its performance”. All seven respondents moderately agreed with the statement: “the system is integrated with my workflow”.

For the following statements, six respondents strongly agreed and one moderately agreed: “the system is easy to use”, “the response time is acceptable”, and “overall, the quality of the system is excellent”.

Four respondents strongly agreed, and three respondents moderately agreed with the statement: “the system features enable me to perform my work well”.

For the statement “the system security is acceptable”, five respondents strongly agreed, one moderately agreed, and one did not answer.

#### Information Quality

Four respondents rated the quality of the information provided by the system as highly acceptable, and three rated it as moderately acceptable.

The distribution of responses to statements regarding information quality were varied. Table 3 presents the results.

**Table 3:** Information quality ratings (n=7).

	Strongly agree	Moderately agree	Moderately disagree	Strongly disagree	Not sure	Not answered
The information is complete	2	5	0	0	0	0
The information is quickly provided	5	1	1	0	0	0
The information is accurate	4	3	0	0	0	0
The information is relevant	7	0	0	0	0	0
The information is available when I need it	4	3	0	0	0	0
The format and layout of the information is acceptable	4	2	1	0	0	0

#### Service Quality

Three of the seven respondents indicated that the quality of the services (ie, technical support and training services) provided for the system were highly acceptable.

For the statement “the implementation process within the Vancouver Island Health Authority catchment area was acceptable”, one respondent strongly agreed, four moderately agreed, and two indicated that they were not sure.

For the statement “the current level of training is acceptable”, four respondents strongly agreed and three moderately agreed.

Three respondents strongly agreed with the statement “the level of on-going support provided is acceptable”, whereas three moderately agreed and one was not sure.

#### System Usage

Users were asked how many times they use the system in a typical day. Four respondents indicated that they used the system constantly throughout the day. The other three respondents indicated that they used the system two to three times per day, one time per day, and three or four times per day. One respondent indicated that they used the system only 1 day per week, and did not specify how many times they typically used the system on that 1 day a week.

One respondent indicated that they used the system three or four times a day. Three respondents indicated that they used the system 5 days a week. Another respondent indicated that they used the system 3 days in a week.

Respondents were asked to indicate the percentage of their clients for whom they used the system. Of the six respondents to this question, four indicated that they did not know. Of the two respondents, one indicated 100% of clients and the other respondent indicated 95%.

All seven respondents indicated that they would definitely recommend the system to health care providers at other hospitals or centers.

Four respondents indicated that they would, given a choice, moderately increase their future use of the system. Two indicated that they would significantly increase use, and one respondent indicated that they would remain with the same use.

#### Other Comments

Two respondents provided additional comments in the survey. The comments related to future desired and planned functionality. One respondent indicated that the new business intelligence system connected to the SDI system would provide more progressive reporting in the future. Connection with Electronic Medical Records (EMRs) or “health networks” was also indicated as desired future functionality.

### Community Health Provider Feedback Survey

Of the 22 community health providers invited to take the survey, 11 individuals responded. Responses to questions that did not relate to the teleopthalmology screening clinics, but rather current staffing complements, care delivery models and service provisions, are not included in this paper. These questions were included to inform ongoing development and deployment of teleopthalmology services.

With regard to being satisfied with how the retinal screening clinics were conducted in their communities, four of the respondents strongly agreed and six agreed.

Satisfaction with various aspects of how the ITHA retinal screening clinics was measured. Most were very satisfied or satisfied with all aspects, with very few neutral responses. No respondents were dissatisfied or very dissatisfied with any of the aspects. Results from the eight respondents to this question are summarized in Table 4, expressed in the number of responses per rating and aspect.

The survey listed potential areas for improvement for the teleopthalmology screening clinics. Two respondents cited that clinic scheduling could be improved. Communication with ITHA, length of clinics, and sharing of client results were each identified by one respondent as potential areas of improvement.

Six of seven respondents to the question about whether ITHA retinal screening clinics helped to raise awareness of diabetes self-management among participating community members indicated that they neither agreed nor disagreed. One respondent agreed. Four respondents skipped this question.

No respondents indicated any problems occurring during retinal screening clinics.

**Table 4:** Satisfaction ratings for retinal screening clinics (n=11).

	Very satisfied	Satisfied	Neutral	Dissatisfied	Very dissatisfied
Communication with ITHA	1	8	0	0	0
Clinic scheduling	1	5	1	0	0
Clinic setup	2	5	0	0	0
Communication with clients	3	4	0	0	0
Professional conduct of team	4	3	0	0	0
Length of clinic	1	6	0	0	0
Clinic dismantling	2	3	2	0	0
Follow-up with client results	3	3	1	0	0

### Operational Cost Measurement

Costs were calculated using the assumption that teleopthalmology removes the need to have referrals from a general practitioner (GP) or ophthalmologist.

#### Traditional versus Teleophthalmology Cost Comparisons

The average per client total cost for traditional ophthalmology screening, according to MSP fees is CAN $647.15 ($92.10 for the specialist exam fee, $160.17 for the GP referral, and $182.92 for the ophthalmologist referral). The average per client travel cost for those clients who would have had to travel to see a specialist for a retinal scan was estimated to be $211.96. This figure was provided by the NIHB program, which provides payment for client travel. This amounts to a total cost per client of $647.15 for the traditional ophthalmology model.

The average per-client cost of teleopthalmology was CAN $590.81. A cost of $553.15 was incurred per client, according to ITHA general ledger entries for teleopthalmology operations. This included the salaries of the teleopthalmology team, travel costs, and medical supplies. In addition, the cost for an ophthalmologist to grade a retinal screen for the teleopthalmology project was $37.66. (No MSP fee codes had yet been created for teleopthalmology grading. A fixed rate of $37.66 per scan was set at the outset of the project. The MSP fee code application was beyond the scope of the teleopthalmology project.)

The direct per-client cost of teleopthalmology was CAN $56.34 less than the direct per-client cost of traditional ophthalmology screening.

## Discussion

### Principal Findings

The teleopthalmology project nearly achieved its goal of screening 600 clients in the first year through the delivery of the clinics via a single mobile unit and limited resources. One of the practical benefits of the teleopthalmology project was the establishment of a rough baseline of those individuals who were either at risk of diabetes or suffer from type 1 or type 2 diabetes. ITHA will now be able to use this baseline to monitor these individuals and provide additional preventative services in an effort to minimize complications and long-term detrimental effects.

The 140 clients who were referred by grading physicians were diagnosed with conditions (ie, cataracts, glaucoma, hypertension, macular degeneration, and other vascular diseases) that may have gone undetected and untreated for long or indefinite periods of time. In addition to the direct cost savings measured for this project, the savings of various indirect costs of worsened and further-progressed conditions (which may also be associated with additional co-morbidities) are possible benefits of the project. Many of the clients screened (and referred) may not have otherwise received care if it had not been made accessible to them via the teleopthalmology project.

Overall user satisfaction with the teleopthalmology system was high. System quality, information quality, and service quality all received favorable ratings.

User experiences with the teleopthalmology system were positive. Respondents planned to use the system no less than before or at the time of the survey. All seven respondents indicated that they would definitely recommend the system to health care providers at other hospitals or centers.

Providers in the First Nations communities rated the setup and operations of the clinics favorably. No dissatisfaction with any aspects of the clinics was indicated. A higher rate of participation from these community health providers would have been desirable to obtain a representative and comprehensive assessment of the teleopthalmology clinics.

The direct average cost savings per client was calculated to be CAN $56.34. The figures used in this evaluation underestimate the cost savings that teleophthalmology offers on an ongoing basis. The main reason is that the capital costs of the teleopthalmology project were included in the analysis; these initial costs were not intended to result in a return on investment in monetary terms nor were they limited to the timeframe of the project and evaluation. The setup cost for relevant components of the traditional health care system was not feasible or included for comparison. As well, the project now operates as an ongoing program, which benefits from greater economies of scale and efficiencies identified throughout the project. Importantly, teleopthalmology was not intended to be a cost-saving project or program, but was intended to improve quality of and access to health care services for First Nations communities.

Although not assessed in this evaluation, there are additional intangible and health-related benefits to clients and caregivers associated with the elimination of travel costs and increased access to screening services (which may prevent the progression of eye-related and other conditions that have impacts in terms of cost, client health and wellness, and caregiver burden). Other potential and indirect benefits that were not assessed include savings in terms of environmental and ecological impacts associated with travel to access health care services. Although not measured in this project, non-monetary impacts on client and caregiver travel such as physical exertion and stress are potential benefits to teleopthalmology.

Demonstrating support for First Nations youth contributed to the successful acceptance of the retinal screening clinics in First Nations communities. The project has also supported capacity building among First Nations people. The two technicians were of First Nations descent; they have gone on to pursue formalized accredited training as ophthalmic assistants.

### Limitations

The evaluation of the teleopthalmology project involved a limited focus on teleopthalmology operations, usage, and costs. Future study could explore the impact of teleopthalmology on local and regional health systems as well as health outcomes of First Nations clients.

A low survey response rate from community health providers yielded a limited understanding of communities’ experiences with the teleopthalmology project.

### Post-Evaluation and Current Activity

Following the initial year of operations, the teleopthalmology project became an official program.

At this time, the teleopthalmology program serves 50 of the 51 First Nations communities on Vancouver Island, with approximately 1300 active clients. The teleophthalmology program now focuses on serving diagnosed diabetics, based on a synthesis of evidence that did not find a clinical benefit of retinal screening for non-diabetic clients [11].

There are now two mobile clinics that provide teleopthalmology services across Vancouver Island. Currently, the referral rate is 19.64% (down from 26.7% in the evaluation period). This may result from a reduced need for treatment and stabilization of health outcome.

The teleopthalmology program receives ongoing funding by the Aboriginal Diabetes Initiative (this is now received through the recently established First Nations Health Authority (FNHA) in BC, which was previously First Nations Inuit Health). The FNHA was established in 2013 as the first Canadian provincial health authority responsible for the planning, designing, management, delivery, and funding of First Nations health programs on a provincial level.

With time, the actual cost savings for the teleopthalmology program have increased compared to the cost savings measured in the teleopthalmology project. Improvements in clinic scheduling have led to the increase in the number of clients that receive retinal screening per clinic. Related to this, improved planning of clinics have contributed to reduced travel costs as multiple clinics are delivered per trip.

Moving forward, additional program costs may decrease with the potential successful establishment of an MSP fee code for Telehealth services that would reimburse grading physicians for their services. Currently, the FNHA provides this funding for grading physicians. During the teleopthalmology project, before the FNHA was established, the federal government via First Nations and Inuit Health (FNIH) provided this funding.

Empowering First Nations youth, in particular those with a willingness to reconnect with their peoples, has been a major point of success with the teleopthalmology program and continues to be a goal of the program. First Nations youth are considered to be uniquely qualified to engage with communities, as they understand the cultural protocols and landscape. The teleopthalmology program ensures that job postings reach youth in First Nations communities. It targets First Nations youth training centers, community events such as health and career fairs, and university First Nations study programs.

## Multimedia Appendix 1

System and use survey.

## Multimedia Appendix 2

Community health provider feedback survey.

## Multimedia Appendix 3

Patient screening and referral volumes by First Nations community.

## Abbreviations

EMR: electronic medical record
FNHA: First Nations Health Authority
FNIH: First Nations Inuit Health
ITHA: Inter Tribal Health Authority
MSP: medical services plan
NIHB: non-insured health benefits
OCAP: Ownership Control Access Possession protocol
OCT: optical coherence tomography
VIHA: Vancouver Island Health Authority
