# Supplementary material for: Teleophthalmology for First Nations Clients at Risk of Diabetic Retinopathy: A Mixed Methods Evaluation
Source: JMIR Med Inform. 2015 Feb 23;3(1):e10. doi: 10.2196/medinform.3872 (PMC4376131; doi:10.2196/medinform.3872)
Supplement: Supplementary file 1 [file medinform_v3i1e10_app1.pdf]

Appendix 1 – System and Use Survey

Canada Health Infoway Inforoute Santé du Canada

System and Use Survey - ITHA TeleOphthalmology

The Inter Tribal Health Authority (ITHA) and Canada Health Infoway (CHI) are conducting a benefits evaluation study in order to improve the quality of the information provided by the health information systems, as well as, the level of satisfaction amongst end-users.

Your feedback and assistance with this survey will help ITHA and CHI to develop better systems and deliver better services.

The following survey consists of specific questions on: the ease and functionality, information quality, service quality related to ITHA TeleOphthalmology Project that has been delivering retinal screening services through ITHA to the First Nations communities within the Vancouver Island Health Authority catchment area, i.e. Vancouver Island and Powell River.

The survey will take approximately 10-15 minutes to complete. Please indicate the response that best represents your opinion. Information that is collected during this survey will be kept anonymous and confidential.

If you have any questions about the survey, please contact Barbara Gray-Wiksten at bgwiksten@xplornet.ca.

Thank you in advance for your participation,

Canada Health Infoway / Inter Tribal Health Authority

Next

Section 1: Overall User Satisfaction

1. In general, how satisfied are you overall with the system you are currently working with? By "system" we mean, the ease and functionality of the system itself, the quality of the information given and the quality of the services provided for the system.

- ○ Highly satisfied
- ○ Moderately satisfied
- ○ Neither satisfied nor dissatisfied
- ○ Moderately dissatisfied
- ○ Not at all satisfied

2. Please indicate your level of agreement or disagreement with each of the following statements below.

	Strongly Agree	Moderately Agree	Moderately Disagree	Strongly Disagree	Not Sure	Not Applicable
a. The system improves my productivity	○	○	○	○	○	○
b. The system improves the quality of care I can provide	○	○	○	○	○	○
c. The system makes my job easier	○	○	○	○	○	○
d. The system enhances our ability to coordinate the continuity of care	○	○	○	○	○	○
e. The system improves our sharing of patient information amongst providers	○	○	○	○	○	○
f. The system enhances the efficiency of ordering retinal screening, referring patients for onward treatment patient follow-	○	○	○	○	○	○

up, identification of diabetic retinopathy, etc.						
g. The alerts, reminders and order set features (i.e. support tools, reporting) improve the quality of my decision-making	○	○	○	○	○	○

3. Are there aspects of the system that you would change, and if so, which ones would they be? Please describe your comments.

4. Do you have any experiences with the system where it has supported the provision of care? Please describe your comments.

Section 2: System Quality

5. Based on your experiences to date with the system, how acceptable is the quality of the system itself (as described by the specific characteristics listed below)? Would you say it is:

○ Highly acceptable ○ Moderately acceptable ○ Neither acceptable nor unacceptable ○ Moderately unacceptable ○ Not at all acceptable

6. Please indicate your level of agreement or disagreement with each of the following statements below.

	Strongly Agree	Moderately Agree	Moderately Disagree	Strongly Disagree	Not Sure
a. The system is easy to use	○	○	○	○	○
b. The response time is acceptable	○	○	○	○	○
c. The system is integrated with my workflow	○	○	○	○	○
d. The system security is acceptable	○	○	○	○	○
e. The system features enable me to perform my work well	○	○	○	○	○
f. The system is reliable in its performance	○	○	○	○	○
g. Overall, the quality of the system is excellent	○	○	○	○	○

Next

Section 3: Information Quality

7. In general, when thinking about the quality of the information provided by the system, do you find the quality of the information to be:

○ Highly acceptable ○ Moderately acceptable ○ Neither acceptable nor unacceptable ○ Moderately unacceptable ○ Not at all acceptable

8. Please indicate your level of agreement or disagreement with each of the following statements below.

	Strongly Agree	Moderately Agree	Moderately Disagree	Strongly Disagree	Not Sure
a. The information is complete	○	○	○	○	○
b. The information is quickly provided	○	○	○	○	○
c. The information is accurate	○	○	○	○	○
d. The information is relevant	○	○	○	○	○
e. The information is available when I need it	○	○	○	○	○
f. The format and layout of the information is acceptable	○	○	○	○	○

Section 4: Service Quality

9. In general, when thinking about the quality of the services (i.e. technical support and training services) provided for the system, do you find the quality of these services to be:

○ Highly acceptable ○ Moderately acceptable ○ Neither acceptable nor unacceptable ○ Moderately unacceptable ○ Not at all acceptable

10. Please indicate your level of agreement or disagreement with each of the following statements below.

	Strongly Agree	Moderately Agree	Moderately Disagree	Strongly Disagree	Not Sure
a. The implementation process within the Vancouver Island Health Authority catchment area was acceptable	○	○	○	○	○
b. The current level of training is acceptable	○	○	○	○	○
c. The level of on-going support provided is acceptable	○	○	○	○	○

Section 5: Public Health Surveillance Specific

- This question to be completed by Public Health Surveillance Personnel Only -

11. Please indicate your level of agreement or disagreement for each of the following statements below.

	Strongly Agree	Moderately Agree	Moderately Disagree	Strongly Disagree	Not Sure
a. The system improves the detection and management of reportable diseases	○	○	○	○	○
b. The system improves the management of immunization process	○	○	○	○	○

Next

Section 6: System Usage

12. In a typical day, how many times do you "use" the system?

- ◯ Number of times per day [ ]
- ◯ Constantly
- ◯ Never

13. In a typical week, please indicate the number of days in which you use the system:

Number of days, per week [ ]

14. Please estimate for what percent of your patients you use the system:

Percentage (enter number or "don't know") [ ]

15. How likely are you to recommend the system to other healthcare providers at other Hospitals or Centres?

◯ Definitely ◯ Probably ◯ May or may not ◯ Probably not ◯ Definitely not

16. Given a choice, would you like to increase or decrease your future use of the system that you are currently working with? Would that be a significant or moderate increase / decrease, or would you like your future use to stay the same?

◯ Significant Increase ◯ Moderate Increase ◯ Moderate Decrease ◯ Significant Decrease ◯ REMAIN THE SAME

Section 7: Other Comments

17. Do you have any other comments you would like to make regarding the system?

[ ]

Next

Section 8: Demographic Information

18. What is your profession?

- ○ Administrative support staff
- ○ Imaging technologist
- ○ Laboratory technician
- ○ Nurse
- ○ Family physician
- ○ Specialist physician (please specify) ______
- ○ Other (please specify) ______

19. How would you describe your "use" of the system? (Check all that apply)

- ☐ I use the system for clinical decision making
- ☐ I use the system to both access patient information and in clinical decision making
- ☐ I use the system to access patient information and support the clinical decision maker

20. How long have you been using the system?

○ Less than a month ○ 1-3 months ○ 4-6 months ○ 7-12 months ○ 1-2 years ○ 3-5 years

21. Currently, how do you receive your patient results?

% FAX ______

% SYSTEM ______

% OTHER (please specify method) ______

22. How would you rate your computer proficiency?

○ None ○ Basic ○ Average ○ Advanced ○ Expert

23. Please check the response(s) that best describe the settings where you work:

- ☐ a. Academic / Teaching Hospital
- ☐ b. Community Clinic / Health Center
- ☐ c. Community Hospital
- ☐ d. Nursing Home / Long Term Care Facility
- ☐ e. Private Office / Clinic
- ☐ f. Other (please specify) ______

24. If you chose a, b, or c to the previous question, do you work within the emergency department?

- ○ Yes
- ○ No

25. Where are you located?

Province/Territory (Click here to choose)

Submit Survey
