# Supplementary material for: Teleophthalmology for First Nations Clients at Risk of Diabetic Retinopathy: A Mixed Methods Evaluation
Source: JMIR Med Inform. 2015 Feb 23;3(1):e10. doi: 10.2196/medinform.3872 (PMC4376131; doi:10.2196/medinform.3872)
Supplement: Supplementary file 2 [file medinform_v3i1e10_app2.pdf]

Appendix 2 - Community Health Provider Feedback Survey

My Surveys Address Book My Account

You have a BASIC account | To remove the limits of a BASIC account and get unlimited questions, upgrade now!

ITHA TeleOphthalmology Program Edit

Edit Survey

Preview Survey

To change the look of your survey, select a theme below.

Blue Ice | Create Custom Theme

+ Add Page

PAGE 1 Edit Page Options ▼ Add Page Logic Move Copy Delete Show this page only

ITHA TeleOphthalmology Program Survey

The ITHA TeleOphthalmology team has been warmly and openly welcomed into your community over this past year and maintains an ongoing commitment to work with you to build a collaborative continuum of diabetic care. ITHA wants to ensure the clinics are a benefit to your community and we would like to ask that you take a few moments to complete this survey on how the TeleOphthalmology Program is working in your community.

There are 10 questions and you can advance to the next question by using the PREV and NEXT buttons at the bottom of the page. There is no need to submit your name or contact information as all responses will be anonymous. Thank you in advance for your kind cooperation.

Please feel free to add any further information that may be relevant, as you know firsthand what your communities have in place and where the TeleOphthalmology Program can provide collaborative support.

+ Add Question ▼

Q1 Edit Question ▼ Add Question Logic Move Copy Delete

1. Overall, I was satisfied with how the retinal screening clinic was conducted in my community.

- Strongly Agree
- Agree
- Neither Agree or Disagree
- Disagree
- Strongly Disagree

Comments

+ Add Question ▼

+ Add Page

PAGE 2 Edit Page Options ▼ Add Page Logic Move Copy Delete Show this page only

+ Add Question ▼

Q2 Edit Question ▼ Add Question Logic Move Copy Delete

2. Does your community have a health care provider that works with ongoing Diabetic care and education?

Yes

No

If yes, please provide the contact name, telephone and/or email address

+ Add Question ▼ Split Page Here

Q3 Edit Question ▼ Add Question Logic Move Copy Delete

3. Is there a standardized Diabetic monitoring program in place?

Yes

No

If yes, please provide details i.e. the type and frequency of monitoring

+ Add Question ▼ Split Page Here

Q4 Edit Question ▼ Add Question Logic Move Copy Delete

4. Is there an individualized care plan in place for each diabetic in your community?

Yes

No

Other (please specify)

+ Add Question ▼ Split Page Here

Q5 Edit Question ▼ Add Question Logic Move Copy Delete

5. Do you have any regular gatherings for community members that focus on diabetes education? Please select as many as applicable.

Elders luncheons

Health and Wellness Programs

Lifestyle Education

Nutrition

Other (please specify)

+ Add Question ▼

+ Add Page

PAGE 3 Edit Page Options ▼ Add Page Logic Move Copy Delete Show this page only

+ Add Question ▼

Q6 Edit Question ▼ Move Copy Delete

6. How satisfied were you with how the ITHA retinal screening clinics were held with respect to each of the following areas.

	Very satisfied	Satisfied	Somewhat satisfied	Very dissatisfied	No comment
Communication with ITHA					
Clinic Scheduling					
Clinic Setup					
Communication with patients					
Professional conduct of team					
Length of clinic					
Clinic dismantling					
Follow-up with patient results					

Other (please specify)

+ Add Question ▼ Split Page Here

Q7 Edit Question ▼ Add Question Logic Move Copy Delete

7. Please check any of the following selections to show us where the TeleOphthalmology retinal screening clinics could improve.

Communication with ITHA

Clinic scheduling

Clinic set-up

Communication with patients

Professional conduct of Team

Length of Clinic

Clinic dismantling

Follow-up with patients

Sharing of patient results

Other (please specify)

+ Add Question ▼

+ Add Page

PAGE 4 Edit Page Options ▼ Move Copy Delete Show this page only

+ Add Question ▼

Q8 Edit Question ▼ Add Question Logic Move Copy Delete

8. In your opinion, did the ITHA retinal screening clinics help to raise awareness of diabetes self management among the community members who participated?

Strongly agree

Agree

Neither agree or disagree

Disagree

Strongly disagree

Other, please describe

+ Add Question ▼ | Split Page Here

Q9 Edit Question ▼ | Move | Copy | Delete

9. Were there any problems that occurred during the retinal screening clinic?

+ Add Question ▼ | Split Page Here

Q10 Edit Question ▼ | Move | Copy | Delete

10. Thank you for taking the time to provide us with your valuable feedback. Please provide us with any comments or suggestions about the clinics to help us serve your community in the best way possible.

+ Add Question ▼

+ Add Page
