# Supplementary material for: Teleophthalmology for First Nations Clients at Risk of Diabetic Retinopathy: A Mixed Methods Evaluation
Source: JMIR Med Inform. 2015 Feb 23;3(1):e10. doi: 10.2196/medinform.3872 (PMC4376131; doi:10.2196/medinform.3872)
Supplement: Supplementary file 3 [file medinform_v3i1e10_app3.pdf]

Appendix 3 – Patient screening and referral volumes by First Nations Community

FIRST NATION	# Patients Screened	# Patient Referrals	% Referred
Ahousaht	27	6	22%
Campbell River	9	0	0%
Cape Mudge	5	0	0%
Chemainus	19	4	21%
Cowichan	1	0	0%
Da’naxda’xw	1	0	0%
Ditidaht	8	2	25%
Dzawada’enuxw	1	1	100%
Ehattesaht	6	2	33%
Gwa'Sala-'Nakwaxda'xw	9	2	22%
Halalt	5	3	60%
Hesquiaht	1	0	0%
Homalco	22	4	18%
Hupacasath	3	0	0%
Huu-ay-aht s	21	4	19%
Ka:'yu:'k't'h'/Che: k: tles7et’h’ s	11	2	18%
Klahoose	1	0	0%
Kwakiutl	23	6	26%
Kwicksutaineuk-ah-kwaw-ah-mish	8	1	13%
Lyackson	5	4	80%
Malahat	8	3	38%
Mowachaht/Muchalaht	9	5	56%
Musqueam	1	0	0%
Namgis	83	22	27%
Nanoose	5	2	40%
Nuuchatlaht	1	0	0%
Pauquachin	7	0	0%
Penelakut	39	13	33%
Qualicum	3	1	33%
Quatsino	18	4	22%
Sliammon	46	10	22%
Snuneymuxw	10	3	30%
Songhees	15	7	47%
Tla-o-qui-aht	8	3	38%
Tlowitsis	1	0	0%
Toquaht	3	0	0%

Tsartlip	17	2	12%
Tsawout	5	1	20%
Tseshaht	13	8	62%
Tseycum	8	0	0%
T'Sou-ke	7	2	29%
Uchucklesaht	3	2	67%
Ucluelet	28	11	39%
Totals	524	140	27%
